# Supplementary material for: Distribution, characteristics, and importance of particulate and mineral-associated organic carbon in China forest: a meta-analysis
Source: PeerJ. 2025 Mar 26;13:e19189. doi: 10.7717/peerj.19189 (PMC11954463; doi:10.7717/peerj.19189)
Supplement: Supplemental Information 1 [file peerj-13-19189-s001.docx]

| **Section and Topic** | **Item #** | **Checklist item** | **Location where item is reported** |
| --- | --- | --- | --- |
| **TITLE** | | |  |
| Title | 1 | Distribution, characteristics, and importance of particulate and mineral-associated organic carbon in China forest | Manuscript 1 |
| **ABSTRACT** | | |  |
| Abstract | 2 | Forests soil organic carbon (SOC) is critical to the global carbon budget, and increasing persistent forest soil carbon storage plays an important role in carbon sequestration. Although the separation of SOC fraction into mineral-associated organic carbon (MAOC) and the particulate organic carbon (POC) can aid in the mechanistic understanding of soil C dynamics, the distribution and drivers of forest soil POC and MAOC are uncharacterized at continental-scale. We collected data related to soil POC, MAOC and total SOC of forest ecosystems in China and analyzed their distribution in forest types and soil depth, as well as influencing factors. MAOC is a major component of SOC content in forests. Both POC and MAOC in forest soils increase with forest age, with mixed forests showing faster growth rates of POC and MAOC compared to monocultures. Meanwhile, the MAOC/SOC ratio decreases as forest age increases, and increases with increasing soil depth, implying the dominance of MAOC in deep SOC content. The MAOC content increases with the increase of SOC content, no upper limit of MAOC was found in the synthesis data. The variability POC content is mainly explained by litter biomass, while MAOC content is mainly associated to microbial biomass carbon content, and they are all minimally affected by the direct effects of climate. Overall, our results emphasize the dominant role of MAOC in China forest SOC pool and its ability to continue increase carbon sequestration in the future. | Manuscript 15 |
| **INTRODUCTION** | | |  |
| Rationale | 3 | MAOC accounts for more than 50% of the total SOC content in forest soils (Xu et al., 2021). In addition, some studies suggest that POC is very important for soil texture, microbial nutrients and energy, and is the main part of soil C component response to climate change (Chaplot and Cooper, 2015; Davidson and Janssens, 2006). The variation of POC and MAOC, and their relative contributions to SOC remain unclear yet. | Manuscript 43 |
| Objectives | 4 | We collected data related to soil POC, MAOC, and total SOC in forest ecosystems in China. Our specific goals were : (1) To determine the distribution of POC and MAOC in forest types, forest age, soil types, and soil depths, and their relative roles in SOC; (2) To isolate the relationship between POC and MAOC and climate, plant and soil factors, and further determine their relative importance. | Manuscript 81 |
| **METHODS** | | |  |
| Eligibility criteria | 5 | To avoid bias in selection of publications and to increase the comparability of data, we followed the PRISMA guide and selected articles that meet the following criteria: (1) The data was directly obtained from field studies in natural forest in China, excluding reviews, modeling studies, and greenhouse experiments; (2) Studies exclusively concerned with the separation of SOC into physical components. We uniformly selected data using the "sodium hexametaphosphate method" (Cambardella and Elliott, 1992) to determine POC (>53 μm) and MAOC (<53 μm) content, to avoid differences in those with soil texture classification methods; (3) The information of soil C component and forest type must clearly been obtained. When multiple publications included the same data from one study, the data were recorded only once; | Manuscript 102 |
| Information sources | 6 | Web of Science (https://www.webofscience.com), Google Scholar (https://scholar. google.com), and China National Knowledge Infrastructure (CNKI, http://www.cnki.net) | Manuscript 102 |
| Search strategy | 7 | The search keywords were “(Particulate organic carbon OR POC, Mineral-associated organic carbon OR MAOC, Particulate organic matter OR POM), Mineral-associated organic matter OR MAOM), (Chinese forest), (Soil aggregates)”, and their combinations. | Manuscript 102 |
| Selection process | 8 | The literature selection process followed the PRISMA guidelines. Two authors (Cheng and Huang) independently screened the titles and abstracts of identified studies against the eligibility criteria. Full-text articles were retrieved for studies meeting the criteria. Any potential disagreements were to be resolved through discussion or, if unresolved, by Cheng, who was designated as the referee. However, no disagreements arose in this study. The screening process was managed using EndNote for citation management and Rayyan for collaborative review. | Manuscript |
| Data collection process | 9 | We used Getdata to collect data from graph and figures.We totally collected 540 observational data from 59 independent studies, which covers major forest ecosystems of China (Fig. S1 and S2). | Manuscript 102 |
| Data items | 10a | The compiled database contained variables including with Soil deepth(cm); Forest type; Latitude(N); Longitude(E); MAT(℃); MAP(mm); Temperature zone; FAO soil group; Aboveground Biomass Carbon (Mg C ha−1); Bulk density; Litterbiomass (g m-2); Living fine root biomass (g·m-2); DOC mg/kg; ROC g/kg; POC(g/kg); MAOC(g/kg); SOC(g/kg); MAOC/SOC; pH; Clay(%); Silt(%); Clay+Silt(%); TN (mg g-1); SOC/TN; MBC (mg/kg); PON(g/kg); MAON(g/kg); TP (g/kg) | Manuscript 123 |
|  | 10b | And we added some parameters from ISRIC-WISE database (<https://data.isric.org>) for those information incomplete sites. The interpolated parameters were mainly used to conduct SEM analysis to determine the driving factors and influencing pathways for POC and MAOC. Based on the soil classification system of the unified FAO UNESCO, we identified eight soil orders (Acrisols, Arenpsols, Cambisols, Ferrarsols, Lithosols, Luvisols, Chernozem, Vertisols) in this study (Figure 4). Due to the limited data for Arenpsols and Vertisols, we did not conduct compared analysis on soil C components of them. | Manuscript 123 |
| Study risk of bias assessment | 11 | The study has conducted a risk of bias assessment on all included studies to ensure their quality and reliability. |  |
| Effect measures | 12 | Specify for each outcome the effect measure(s) (e.g. risk ratio, mean difference) used in the synthesis or presentation of results. | Manuscript |
| Synthesis methods | 13a | In the process of data extraction, it is sometimes impossible to extract MAOC content and POC content directly from the data in the chart, so we use the following formula for conversion:  POCcontent (g/kg) = cPOCconcentration × cPOC proportion + fPOCconcentration × fPOC proportion (1)  POCcontent (g/kg) =cPOCcontent + fPOCcontent (2)  SOCcontent (g/kg) = POCcontent + MAOCcontent (3)  cPOC represents coarse POC and fPOC represents fine POC. | Manuscript 152 |
|  | 13b | Heterogeneity across studies was assessed using the Q statistic and I² values. An I² value greater than 50% indicated substantial heterogeneity. |  |
|  | 13c | A funnel plot was used to assess publication bias. Egger's test was also conducted to evaluate the symmetry of the funnel plot. No significant publication bias was detected. |  |
|  | 13d | Subgroup analyses were conducted to explore the effects of forest type (broadleaf, conifer, and mixed forests), soil depth, and forest age on POC and MAOC content. This analysis aimed to identify variations among groups and their potential drivers. |  |
|  | 13e | Sensitivity analyses were performed by excluding studies with incomplete soil depth data or extreme outliers to evaluate the robustness of the results. |  |
|  | 13f | Reporting bias and methodological quality were assessed using the Cochrane Risk of Bias tool. Results showed minimal bias across included studies. |  |
| Reporting bias assessment | 14 | Reporting bias was assessed using funnel plots and Egger's regression test. The results indicated no significant evidence of bias, suggesting the robustness of the study synthesis. |  |
| Certainty assessment | 15 | No formal certainty assessment (e.g., GRADE) was conducted due to the variability in data sources and the exploratory nature of this study. However, a qualitative assessment of study quality and consistency was performed to ensure reliability. |  |
| **RESULTS** | | | Manuscript 183-235 |
| Study selection | 16a | PRISMA flow diagram (Fig. S1) showed the result of study selection, We have neglected some research on atypical forests that are restored from the degraded arid lands. After multiple screening of articles, we totally collected 540 observational data from 59 independent studies, which covers major forest ecosystems of China. 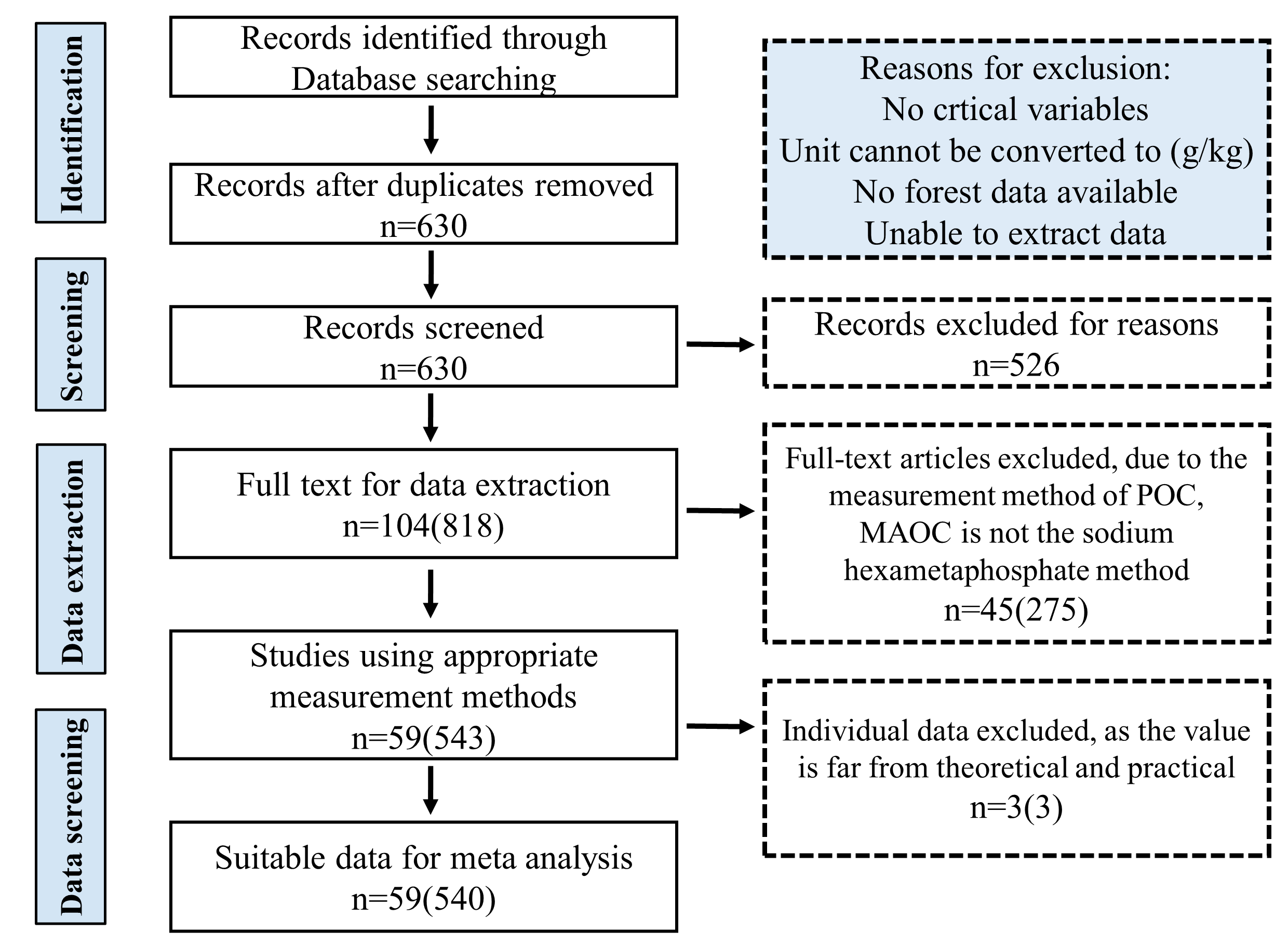 | Supplementary file |
|  | 16b |  |  |
| Study characteristics | 17 | Cite each included study and present its characteristics.  See details at https://zenodo.org/records/13992794.   \| Reference number \| **Citation** \| Latitude(N) \| Longitude(E) \| MAT(℃) \| MAP(mm) \| \| --- \| --- \| --- \| --- \| --- \| --- \| \| 1 \| Tian et al,2020 \| 29.767333 \| 110.0874 \| 10.7 \| 2100 \| \| 2 \| Han et al,2017 \| 36.778333 \| 109.229444 \| 8.8 \| 510 \| \| 3 \| Yang et al,2021 \| 30.383333 \| 119.883333 \| 17 \| 1854 \| \| 4 \| Hu et al,2021 \| 27.55 \| 105.366667 \| 21.8 \| 1607 \| \| 5 \| Xu et al,2021 \| 29.15 \| 118.416667 \| 16.4 \| 1814 \| \| 6 \| Lan et al,2021 \| 25.653611 \| 105.6 \| 18.4 \| 1100 \| \| 7 \| Geng et al,2020a \| 26.744167 \| 105.059167 \| 17.9 \| 1475 \| \| 8 \| Peng et al,2023 \| 23.5 \| 103.066667 \| 19.8 \| 800 \| \| 9 \| Liao et al,2022 \| 27.166667 \| 100.166667 \| 18.2 \| 965 \| \| 10 \| Chen et al,2020a \| 27.7 \| 117.75 \| 17.5 \| 1900 \| \| 11 \| Yang et al,2023 \| 36.003889 \| 109.015556 \| 9 \| 577 \| \| 12 \| Hu et al,2022 \| 27.55 \| 104.766667 \| 21.8 \| 1607 \| \| 13 \| Li et al,2022 \| 23.15 \| 112.516667 \| 21 \| 1900 \| \| 14 \| Wang et al,2019 \| 26.55 \| 118.1 \| 19.4 \| 1817 \| \| 15 \| Xiao et al,2021 \| 24.883333 \| 107.933333 \| 16.9 \| 1675 \| \| 15 \| Xiao et al,2021 \| 23.566667 \| 108.316667 \| 21 \| 1275 \| \| 15 \| Xiao et al,2021 \| 23.566667 \| 108.316667 \| 21.5 \| 1250 \| \| 16 \| Chen et al,2020b \| 39.916667 \| 115.333333 \| 5.4 \| 505 \| \| 17 \| Geng et al,2019 \| 26.746667 \| 115.075833 \| 17.9 \| 1475 \| \| 17 \| Geng et al,2020b \| 26.746667 \| 115.075833 \| 17.9 \| 1475 \| \| 18 \| Sun et al,2023 \| 26.183333 \| 117.466667 \| 19.4 \| 1630 \| \| 19 \| Tong et al,2016 \| 36.85 \| 109.333333 \| 8.8 \| 505 \| \| 20 \| Han et al,2022 \| 48.1875 \| 125.127778 \| 1.3 \| 503 \| \| 21 \| Pan et al,2022 \| 35.166667 \| 115.095833 \| 15.5 \| 609 \| \| 22 \| Zhang et al,2023 \| 33.55 \| 111.916667 \| 15.9 \| 836 \| \| 23 \| Zhang et al,107 \| 44.866667 \| 82.883333 \| 6.8 \| 139 \| \| 24 \| Lu et al,2022 \| 34.4 \| 103.1 \| 6.7 \| 626 \| \| 25 \| Wu et al,2022 \| 35.5 \| 106.383333 \| 5.8 \| 476 \| \| 26 \| Luo et al,2019 \| 23.7 \| 113.616667 \| 10 \| 587 \| \| 27 \| Chen et al,2012 \| 23.166667 \| 112.166667 \| 21.5 \| 1927 \| \| 28 \| Fang et al,2021 \| 25.716667 \| 116.416667 \| 18.3 \| 1730 \| \| 29 \| Dong et al,2023 \| 32.1 \| 114.016667 \| 15.2 \| 1063 \| \| 30 \| Sheng et al,2015 \| 28.416667 \| 113.933333 \| 17.7 \| 1900 \| \| 31 \| Chen et al,2023 \| 25.033333 \| 116.616667 \| 20.1 \| 1600 \| \| 32 \| Liu et al,2023 \| 41.850833 \| 124.9 \| 4.7 \| 800 \| \| 33 \| Tian et al,2015 \| 42.4 \| 128.6 \| 2 \| 700 \| \| 34 \| Zhang et al,2022 \| 21.27 \| 110.54 \| 23 \| 1550 \| \| 35 \| Bai et al,2020 \| 25.620278 \| 105.614167 \| 18.4 \| 1100 \| \| 36 \| Chen et al,2019 \| 29.7 \| 103.233333 \| 16.2 \| 1490 \| \| 37 \| Zhang et al,2023 \| 35.805556 \| 119.772222 \| 12.7 \| 662 \| \| 38 \| Geng et al,2009 \| 40.501209 \| 115.818329 \| 16.2 \| 629 \| \| 38 \| Geng et al,2009 \| 40.647102 \| 117.480438 \| 16.2 \| 629 \| \| 38 \| Geng et al,2009 \| 39.941715 \| 115.604874 \| 16.2 \| 629 \| \| 38 \| Geng et al,2009 \| 40.541252 \| 116.820999 \| 16.2 \| 629 \| \| 38 \| Geng et al,2009 \| 39.974239 \| 116.191803 \| 16.2 \| 629 \| \| 39 \| Zheng et al,2023 \| 23.183333 \| 101.1 \| 19.5 \| 1499 \| \| 40 \| Xiang et al,2022 \| 23.166667 \| 112.166667 \| 21.5 \| 1927 \| \| 41 \| Xiang et al,2022 \| 23.166667 \| 112.166667 \| 21.5 \| 1927 \| \| 42 \| Shi et al,2019 \| 36.4790921 \| 110.0026703 \| 10 \| 513 \| \| 43 \| Fang et al,2021 \| 26.7 \| 115.066667 \| 17.9 \| 1600 \| \| 44 \| Duan et al,2023 \| 25.153056 \| 108.014167 \| 19 \| 1389 \| \| 45 \| Shi et al,2023 \| 35.783333 \| 108.583333 \| 10 \| 587 \| \| 46 \| Zhang et al,2023 \| 22.433333 \| 106.866667 \| 22 \| 1350 \| \| 47 \| Zhang et al,2014 \| 36.216667 \| 103.783333 \| 8.4 \| 263 \| \| 48 \| Qing et al,2015 \| 31.783333 \| 102.7 \| 7.27 \| 678 \| \| 49 \| Liu et al,2022 \| 34.566667 \| 105.633333 \| 10.7 \| 534 \| \| 50 \| Liu et al,2018 \| 31.683333 \| 103.883333 \| 9.3 \| 825 \| \| 51 \| Liu et al,2020 \| 34.806944 \| 108.093056 \| 10.8 \| 602 \| \| 52 \| Zhang et al,2020 \| 34.55 \| 107.9 \| 11.5 \| 592 \| \| 53 \| Deng et al,2020 \| 32.116667 \| 119.2 \| 5.5 \| 1126 \| \| 54 \| Xi et al,2020 \| 28.583333 \| 114.583333 \| 16.2 \| 2100 \| \| 55 \| Shi et al,2014 \| 47.233333 \| 128.8 \| -0.3 \| 676 \| \| 56 \| Liu et al,2023 \| 27.866667 \| 99.65 \| 5.4 \| 620 \| \| 57 \| Li et al,2018 \| 36.716667 \| 111.983333 \| 8.7 \| 600 \| \| 58 \| Han et al,2022 \| 29.833333 \| 103.25 \| 16.8 \| 1400 \| \| 59 \| Yang et al,2009 \| 26.219444 \| 117.433333 \| 19.1 \| 1749 \| | Supplementary file  And    https://zenodo.org/records/13992794. |
| Risk of bias in studies | 18 | Standard deviation. | Manuscript 185 |
| Results of individual studies | 19 | The POC content in forest soils ranged from 0.18 g/kg to 65.33 g/kg, while the MAOC content ranged from 0.61 g/kg to 119.68 g/kg, and the MAOC/SOC value ranged from 0.14 to 99 (Fig. 1a, b and d).Forest type had a significant impact on POC and SOC (Table 1), showing higher POC and SOC in mixed forests than in broad-leaf and conifer forests (Fig. 1a). MAOC accounted for 63% of total SOC across three forest types, showing a constant MAOC/SOC ratio in forest types (Fig. 1d). Both POC and MAOC increased with SOC (Fig. 2a, b), but the slope was steeper for MAOC (Fig. 2b, slope: 0.49; p < 0.001) than for POC (Fig. 2a, slope: 0.51; p < 0.001), suggesting the relative dominance of MAOC in SOC composition at high SOC content. Overall, POC, MAOC, and the MAOC/SOC ratio were not significantly affected by either latitude, climatic factors (MAT, MAP), or above-ground biomass carbon (ABC) (Table 1). |  |
| Results of syntheses | 20a | Forest age significantly influenced POC, MAOC, SOC, and the MAOC/SOC ratio, and the interaction of forest type and forest age was also significant (Table 1). When considering each individual forest type, POC, MAOC, and SOC increased dramatically with increasing stand age in mixed forests (Fig. 3a,b,c), while those in coniferous forests (CF) and broad-leaf forests (BF) increased relatively slowly. On the other hand, the MAOC/SOC ratio in mixed forests decreased as stand age increased (Fig. 3d), due to the relatively faster rate of POC with stand age compared to MAOC. | Manuscript 197 |
|  | 20b |  |  |
|  | 20c |  |  |
|  | 20d |  |  |
| **DISCUSSION** | | | Manuscript 237-342 |
| Discussion | 23a | POC and MAOC have different distributions in forest types and soil depth, and potential being response different to environmental changes. | Manuscript |
|  | 23b | This study has several limitations. First, the data primarily represent forests in eastern China, with limited coverage in western China and Tibet, which may underestimate regional variations in soil carbon dynamics. Second, the focus on surface soils (0–20 cm) limits the understanding of deep soil carbon processes, which are crucial for long-term carbon storage. Third, as most forests studied were secondary forests, the linear relationships between SOC fractions and environmental factors may not fully represent primary forest systems. Future studies should aim to expand the geographic and depth coverage of sampling and include more primary forests for comprehensive analyses. | Manuscript |
|  | 23c |  |  |
|  | 23d |  |  |
| **OTHER INFORMATION** | | |  |
| Registration and protocol | 24a | This work was supported by the National Natural Science Foundation of China (Nos. 32171643, 41671115, U1703332). | Manuscript 350 |
|  | 24b | This systematic review was not pre-registered or guided by a specific protocol due to its exploratory nature. However, all methodologies were designed to align with PRISMA guidelines, ensuring transparency and reproducibility. |  |
|  | 24c |  |  |
| Support | 25 | Authors confirm no conflict of interest. |  |
| Competing interests | 26 | Upon request. |  |
| Availability of data, code and other materials | 27 | Data of this research is available at https://zenodo.org/records/13992794. |  |

*From:*  Page MJ, McKenzie JE, Bossuyt PM, Boutron I, Hoffmann TC, Mulrow CD, et al. The PRISMA 2020 statement: an updated guideline for reporting systematic reviews. BMJ 2021;372:n71. doi: 10.1136/bmj.n71. This work is licensed under CC BY 4.0. To view a copy of this license, visit <https://creativecommons.org/licenses/by/4.0/>
