# Supplementary figures and images for: Distribution, characteristics, and importance of particulate and mineral-associated organic carbon in China forest: a meta-analysis
Source: PeerJ. 2025 Mar 26;13:e19189. doi: 10.7717/peerj.19189 (PMC11954463; doi:10.7717/peerj.19189)

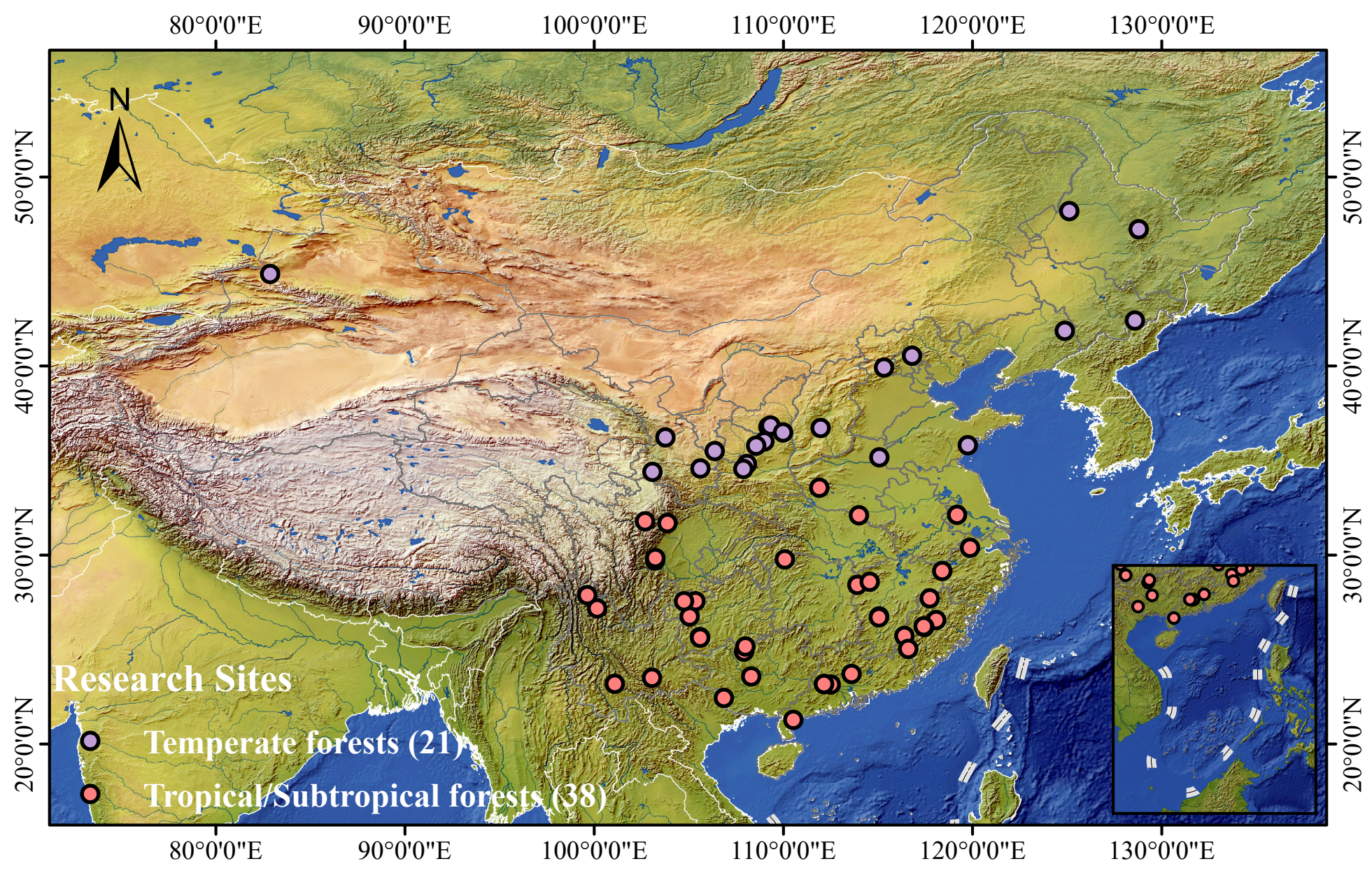

Supplement: Supplemental Information 2 — Our research includes 21 research sites (purple cycles) in temperate forests and 38 research sites (red circles) in tropical/subtropical forests. Light green represents areas with forest cover, while light blue represents areas without forest cover. [file peerj-13-19189-s002.pdf]

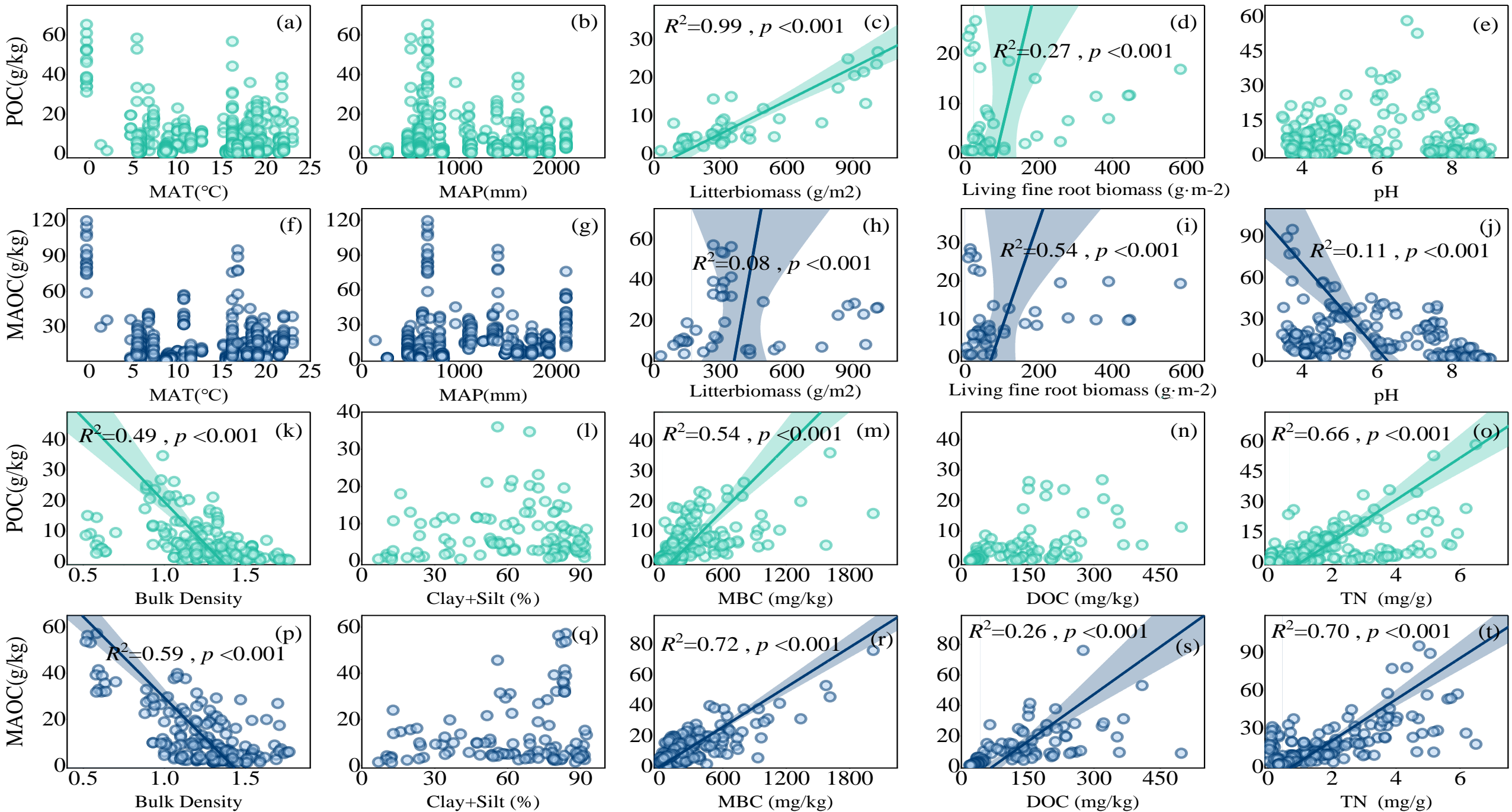

Supplement: Supplemental Information 3 — (a, f) Linear regression of POC and MAOC with MAT, respectively; (b, g) Linear regression of POC and MAOC with MAT, respectively; (c, h) Linear regression of POC and MAOC with litter biomass, respectively; (d, i) Linear regression of POC and MAOC with living fine root biomass, respectively; (e, j) Linear regression of POC and MAOC with pH, respectively; (k, p) Linear regression of POC and MAOC with bulk density, respectively. (l, q) Linear regression of POC and MAOC with clay+silt%, respectively; (m, r) Linear regression of POC and MAOC with MBC, respectively; (n, s) Linear regression of POC and MAOC with DOC, respectively; (o, t) Linear regression of POC and MAOC with TN, respectively; MAT, mean annual temperature; MAP, mean annual precipitation; MBC, microbial biomass carbon; DOC: dissolved organic carbon; TN: total nitrogen. The solid lines indicate significant linear regressions (p < 0.05), and the shaded areas represent 95% confidence intervals. [file peerj-13-19189-s003.pdf]

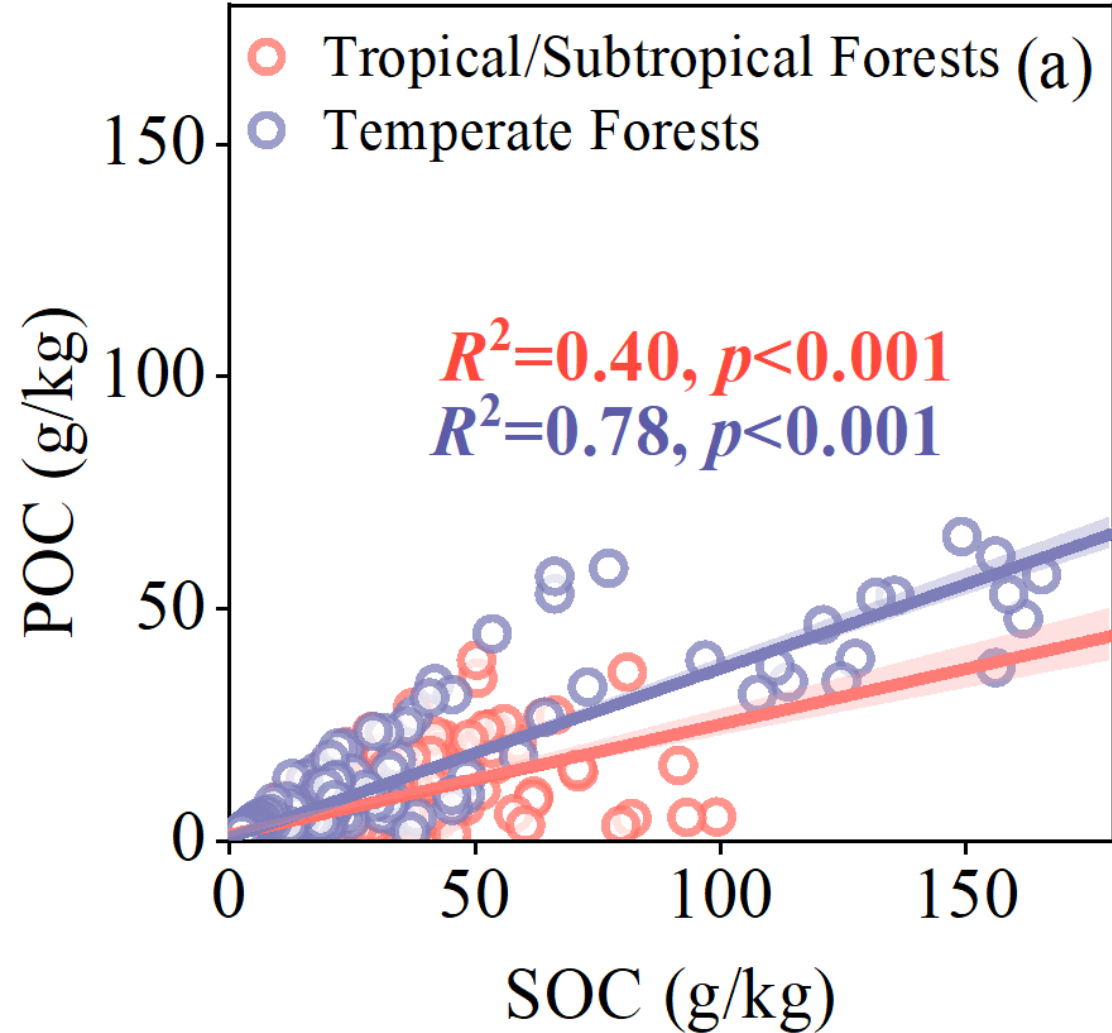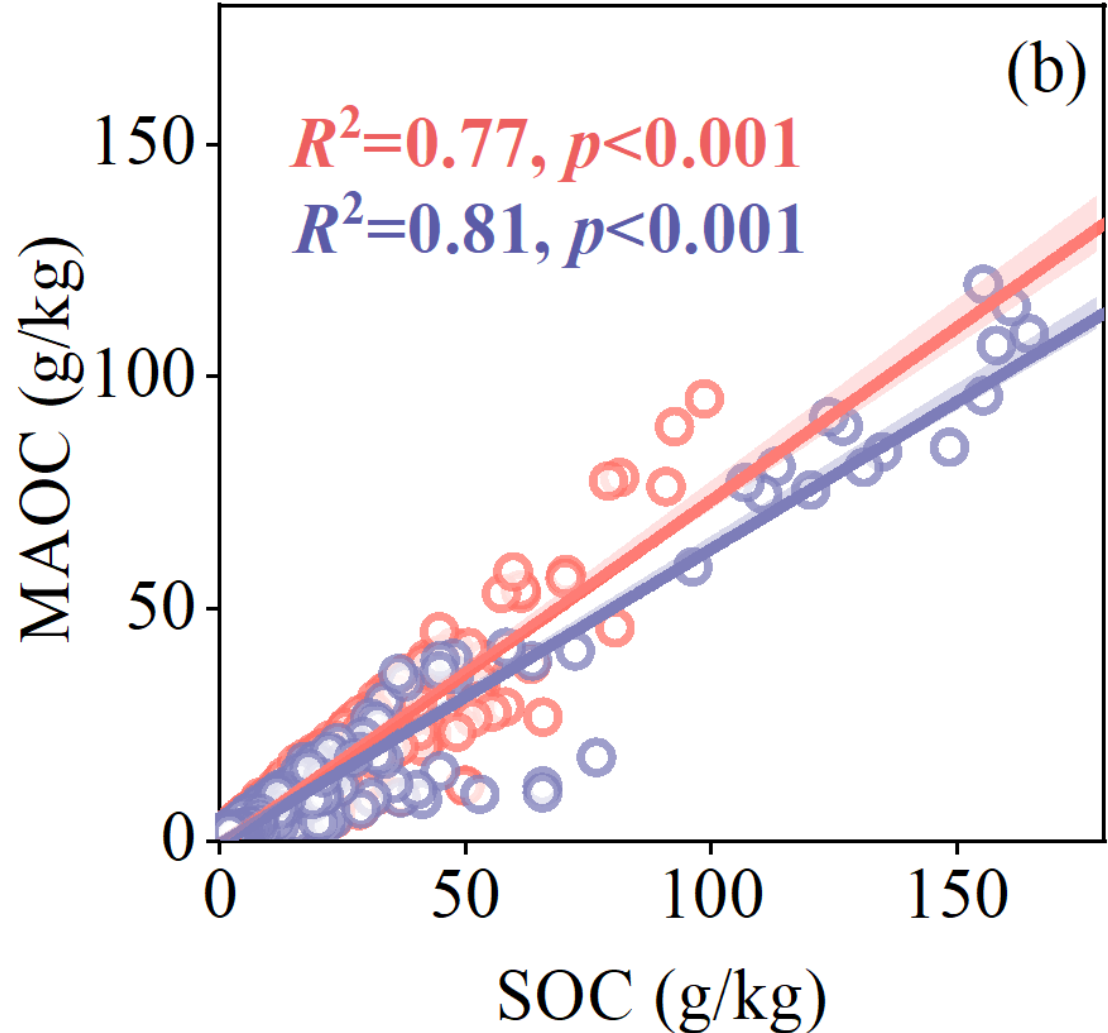

Supplement: Supplemental Information 4 — The purple and red circles represent linear regression for temperate forests and tropical/subtropical forests, respectively. The solid line represents a significant linear relationship (p < 0.05), the shaded area represents a 95% confidence interval. [file peerj-13-19189-s004.pdf]
