# Supplementary material for: Distribution, characteristics, and importance of particulate and mineral-associated organic carbon in China forest: a meta-analysis
Source: PeerJ. 2025 Mar 26;13:e19189. doi: 10.7717/peerj.19189 (PMC11954463; doi:10.7717/peerj.19189)
Supplement: Supplemental Information 5 [file peerj-13-19189-s005.docx]

|  | **Effect** | **t_value** | ***p*** |
| --- | --- | --- | --- |
| POC | SOC | 33.96 | <0.001*** |
|  | Temperature Zone | 0.89 | 0.377 |
|  | SOC:Temperature Zone | -5.99 | <0.001*** |
| MAOC | SOC | 56.34 | <0.001*** |
|  | Temperature Zone | -0.63 | 0.526 |
|  | SOC:Temperature Zone | 4.97 | <0.001*** |
