# Supplementary material for: Distribution, characteristics, and importance of particulate and mineral-associated organic carbon in China forest: a meta-analysis
Source: PeerJ. 2025 Mar 26;13:e19189. doi: 10.7717/peerj.19189 (PMC11954463; doi:10.7717/peerj.19189)
Supplement: Supplemental Information 6 — ABC: above-ground biomass carbon; MAP: mean annual precipitation; MBC: microbial biomass carbon; DOC: dissolved organic carbon; LB: litter biomass; ROC: readily oxidized organic carbon; RB: fine root biomass; MAT: mean annual temperature; BD: bulk density; TN: total nitrogen; TP: total phosphorus. [file peerj-13-19189-s006.docx]

|  | **Predictors** | **Standard coefficient** | **R^2^ adj.** | **P value** |
| --- | --- | --- | --- | --- |
| POC | (Constant) | 2.072 | .187 | 0.203 |
|  | ABC | 0.026 |  | <.001*** |
|  | MAP | -.006 |  | <.001*** |
|  | MBC | 0.011 |  | <.001*** |
|  | DOC | 0.031 |  | 0.002** |
|  | LB | 0.011 |  | 0.026** |
| MAOC | (Constant) | 5.016 | .381 | 0.047** |
|  | ROC | 0.121 |  | <.001*** |
|  | ABC | 0.005 |  | <.001*** |
|  | DOC | 0.016 |  | 0.013** |
|  | RB | 0.005 |  | <.001*** |
|  | MAT | 0.155 |  | <.001*** |
|  | BD | 0.132 |  | <.001*** |
|  | pH | 0.670 |  | 0.010** |
|  | TN | 0.460 |  | <.001*** |
|  | TP | 3.405 |  | <.001*** |
|  | MAP | 0.002 |  | 0.003** |
|  | MBC | 0.004 |  | 0.028** |
| SOC | (Constant) | 7.146 |  | 0.461 |
|  | ABC | 0.006 |  | <.001*** |
|  | ROC | 0.178 |  | <.001*** |
|  | DOC | 0.022 |  | 0.001*** |
|  | MAT | 0.170 |  | <.001*** |
|  | BD | 0.157 |  | <.001*** |
|  | MBC | 0.006 |  | <.001*** |
|  | TP | 4.805 |  | <.001*** |
|  | TN | 0.608 |  | 0.003** |
|  | pH | 0.945 |  | 0.011** |
|  | RB | 0.007 |  | 0.013** |
